# Long-Term Prognosis of Suspected Myocarditis and Cardiomyopathy Associated with Viral Infection of the Myocardial Tissue: A Meta-Analysis of Cohort Studies

**DOI:** 10.1155/2019/9342792

**Published:** 2019-12-16

**Authors:** Wen-Hao Chen, You-Sheng Guo, Dong-Hui Zhang, Huan-Ji Zhang

**Affiliations:** ^1^Graduate Department, Guangdong Medical University, Zhanjiang, China; ^2^Cardiovascular Center, The Eighth Affiliated Hospital, Sun Yat-Sen University, Shenzhen, China

## Abstract

**Aim:**

Myocarditis and cardiomyopathy impose a substantial economic burden on society. Many studies have examined the effects of various predictors on the prognosis of these diseases, such as the left ventricular systolic function, the New York Heart Association glomerular filtration rate, the QT interval, and the presence of viruses. In the present study, we conducted a meta-analysis of cohort studies to investigate the significance of the presence of viruses in the myocardial tissue on the prognosis of these diseases.

**Methods:**

The Embase, PubMed, and Cochrane library databases were searched for relevant literature that had been published between January 1, 1964 and August 14, 2018. The inclusion criteria were patients over 18 years of age, suspected myocarditis or dilated cardiomyopathy, accepted myocardial biopsy, and the detection of virus in the myocardial tissue.

**Results:**

In total, 10 studies met the inclusion criteria. These studies included 1006 patients with suspected myocarditis or idiopathic heart disease for whom the primary endpoint was all-cause death, heart transplant, or re-hospitalization due to fatal arrhythmia and heart failure. There was no significant difference in the prognosis of virus-positive and virus-negative patients with myocarditis or dilated cardiomyopathy confirmed by endomyocardial biopsy (EMB) [hazard ratio (HR) = 1.40, 95% confidence interval (CI) = 0.93–2.12, *P* = 0.11]. However, virus-negative patients had a better prognosis following nonspecific treatment (HR = 1.40, 95% CI = 1.06–1.86, *P* = 0.02) and right ventricular biopsy (HR = 2.08, 95% CI = 1.07–4.04, *P* = 0.03).

**Conclusions:**

The presence of a virus did not worsen the long-term prognosis of patients with suspected myocarditis or dilated cardiomyopathy. However, virus-positive patients who did not undergo specific treatment or who underwent right ventricular biopsy did have a worse prognosis. Thus, the early diagnosis of the presence of viral infection in the myocardium will improve the prognosis of patients.

## 1. Introduction

The application of endomyocardial biopsy (EMB) has shown that some nonischemic heart failures are associated with myocardial disease, among which myocarditis and dilated cardiomyopathy are the most common. Although the pathogenesis of nonischemic cardiomyopathy remains unclear, it is known that these diseases are most commonly caused by microbial infections (e.g., viruses) and autoimmune diseases [1], with physical and chemical factors (e.g., alcohol) and the side effects of drugs, also contributing to a lesser extent.

It has recently been shown that viral populations in the myocardium are constantly changing [2] and our understanding of the pathophysiological processes that lead to myocardial disease are gradually improving. In a comparison of patients before and after treatment through the performance of a second myocardial biopsy, Kuhl et al. [3, 4] found that the cardiac function showed greater recovery in patients who changed from virus-positive to virus-negative than in those who continued to be virus-positive, and so considered that the persistence of the virus was a predictor of prognosis. However, to the best of our knowledge, there has been no meta-analysis or systematic review of the impact of viruses on the prognosis of myocardial disease and no assessment of whether cardiomyocytes contribute to the assessment of clinical outcomes.

The purpose of this study was to examine the role viruses play in myocarditis and dilated cardiomyopathy, and to evaluate whether the presence or absence of viruses has potential value as a predictor of the nontransplant survival rate.

## 2. Materials and Methods

### 2.1. Search Strategy and Selection Criteria

Prospective and retrospective cohort studies relating to suspected myocarditis or idiopathic cardiomyopathy, myocardial biopsy, and the detection of viruses that were published between January 1, 1964 and August 14, 2018 were searched for in Embase, PubMed, and the Cochrane library using the search keywords “congestive cardiomyopathy,” “dilated cardiomyopathy,” “cardiomyopathy,” “myocarditis,” “carditis,” “myocardial biopsy,” “endomyocardial biopsy,” “cardiac biopsies,” and “heart muscle biopsy.” The literature search was undertaken independently by two of the authors (Wen-Hao Chen and You-Sheng Guo) and citation searches were also made on related articles to ensure a complete literature search had been undertaken. The full text of each article that was retrieved was examined by two reviewers (Huan-Ji Zhang and Dong-Hui Zhang) to determine whether the study met the inclusion criteria (see below). A review of the full text and the final selection of articles for inclusion in this study was then completed by one of the authors (Wen-Hao Chen).

### 2.2. Inclusion and Exclusion Criteria

Research needed to meet the following criteria for inclusion in this study: (1) cohort study; (2) patients over 18 years of age with suspected myocarditis or dilated cardiomyopathy; (3) accepted myocardial biopsy and detection of a virus in the myocardial tissues.

Any study that met one or more of the following criteria was excluded: (1) nonenglish literature, conference summary, or case report; (2) patients less than 18 years of age, with coronary heart disease (coronary stenosis > 50%) or no myocardial biopsy; (3) heart transplant or clear pathogenesis factors (e.g., cardiac amyloidosis, peripartum cardiomyopathy, human immunodeficiency virus (HIV) infection, systemic lupus erythematosus); (4) no virus-negative control group or an inability to extract key data.

The main outcomes were death, heart transplantation, and hospitalization due to fatal arrhythmia or heart failure. If a study reported both cardiovascular-related deaths and all-cause death endpoints, the former was preferred.

### 2.3. Quality Assessment

The Newcastle-Ottawa scale (NOS) was applied to all studies that met the inclusion criteria. The NOS assesses studies based on three major elements (selection, comparability, and outcome), which can attain maximum scores of 4, 2, and 3 stars, respectively, to give an overall maximum score of 9 stars [5]. It is generally considered that a study needs to attain a score of >6 stars to be considered high quality. The NOS is highly versatile and reliable and is often recommended for evaluating the quality of cohort studies by the Cochrane Handbook for Systematic Reviews of Interventions Version 5.1.0 [6]. The scoring process was completed by two of the authors (Dong-Hui Zhang and Huan-Ji Zhang). When there was inconsistency in the quality evaluation, another author (Wen-Hao Chen) decided which score to adopt.

### 2.4. Data Extraction

The following data were extracted from each of the articles that was included in this study: the first author's name, year of publication, country, study design, following period, groups and number of patients, age, initial diagnosis, methods of virus detection and viral types, outcomes, and any information about the article's quality. Survival data were usually provided as the heart transplant-free survival rate. In addition, the hazard ratio (HR), *p*-value, Kaplan–Meier survival curve, and 95% confidence interval (95% CI) were obtained from related articles. Where results from the same study for a particular risk factor had been published in more than one manuscript, the paper with the most complete data was used or, where the data were the same, the results from the most recent publication were used. This process was completed by You-Sheng Guo.

### 2.5. Data Synthesis

The results are presented as the log hazard ratio (logHR) and the standard error (SE), which in some cases could be extracted directly from the articles. Where no specific data were provided for the HR and 95% CI, the logHR and SE were calculated by extracting Kaplan–Meier survival curves using Engauge Digitizer version 10.1 following Tierney's software guidance [7].

Heterogeneity was defined as *I*^2^ > 50% or *P* < 0.10. A fixed effects model can be used where no heterogeneity is detected, whereas a random effects model should be used where heterogeneity is statistically significant. The weight of each study was calculated using the inverse variance method and was adjusted in the effects model. Outcomes for the virus-positive and virus-negative groups were compared by examining the HR and 95% CI: if HR was >1 and the 95% CI did not contain 1, then the two groups were significantly different, with the virus-positive group having a poorer outcome than the virus-negative group. All of the above analyses were presented using RevMan5.3. In addition, publication bias was examined by performing Egger's test in Stata12.

## 3. Results

### 3.1. Characteristics of the Selected Studies

**Figure 1:**
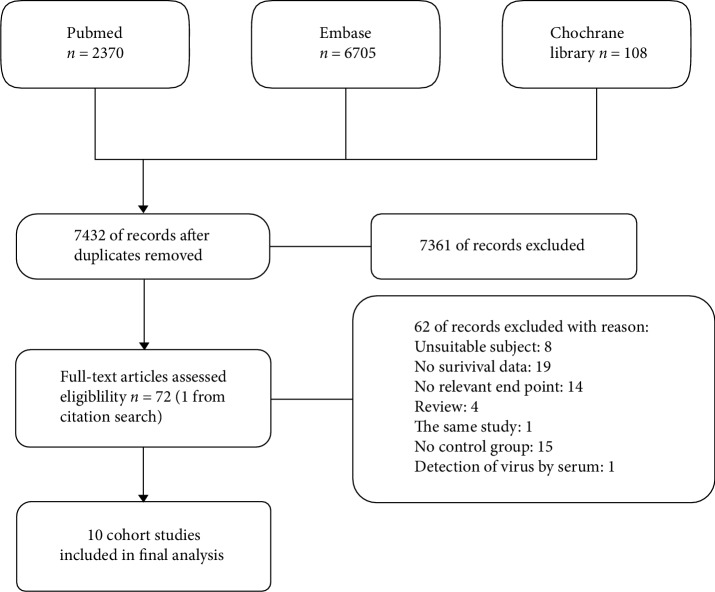
Process for inclusion of eligible documents.

In total, we scanned 9183 articles from PubMed, Embase, and the Cochrane library, 9111 of which were excluded as a result of the removal of duplicates and record screening (no mention of a virus, case report, review, comment, conference abstract, nonenglish publication, related to children, heart transplantation, HIV infection, peripartum cardiomyopathy). Full-text review resulted in a further 62 articles being excluded due to an unsuitable subject, no relevant endpoint, no control group, or no survival data or because they were a review or from the same study as another report. This resulted in 10 articles that included 1006 patients with suspected myocarditis or dilated cardiomyopathy confirmed by EMB being selected for inclusion in the meta-analysis: (1) Why et al. [8], (2) Figulla et al. [9], (3) Fujioka et al. [10], (4) Caforio et al. [11], (5) Kindermann et al. [12], (6) Nowalany-Kozielska et al. [13], (7) Tebbe et al. [14], (8) Karatolios et al. [15], (9) Kuethe et al. [16], and (10) Hjalmarsson et al. [17]. The screening process is shown in Figure 1 and the characteristics of the selected studies are provided in Tables 1 and 2.

The quality scores of the 10 studies ranged from 7 to 9 (Table 3), indicating that they were all high-quality studies according to the NOS. Only 3 of the 10 studies provided HR values and 95% CIs. Therefore, we used Tierney's method [17] (as recommended by the Cochrane Handbook) to extract the number of people, the number of events, and the Kaplan–Meier curve from each article, allowing us to calculate the HR value and its interval. Five of the studies were from Germany, while one was from each of Japan, Italy, the United Kingdom, Poland, and Switzerland, and none of the studies were included in other publications. The sample sizes ranged from 26 to 293 patients, with a total of 440 patients in the virus-positive group and 566 patients in the virus-negative group. The shortest follow-up period was only 6–12 months [9], while the longest was 112 ± 57 months [16].

Regarding the baseline data from the 10 cohort studies, Figulla et al. [9] reported that atrial fibrillation was more common in the virus-positive group than in the virus-negative group (35% vs. 14%, respectively; *P* = 0.04) and that the myofibril volume fraction also significantly differed between the two groups (57.5 ± 4.3 vs. 55.1 ± 3.1, respectively; *P* = 0.036). Furthermore, Caforio et al. [11] reported that clinical left heart failure and right heart failure were more common in the virus-positive group than in the virus-negative group (*P* = 0.005 and 0.01, respectively). However, there were no statistically significant differences between the groups in any of the other cohort studies. Only Kindermann et al. [12] reported cardiac mortality and all-cause mortality as the primary endpoint (priority of cardiac-related deaths), with all other studies reporting all-cause mortality and heart transplantation.

### 3.2. Long-Term Prognosis of Virus-Positive Patients

**Figure 2:**
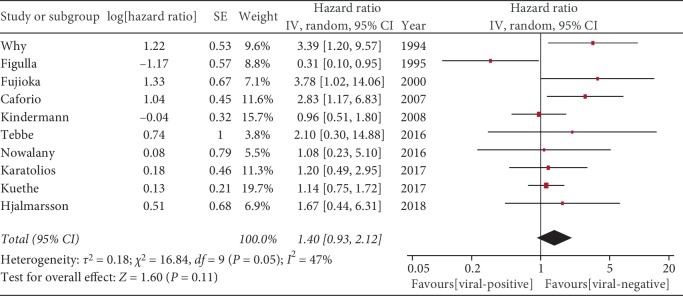
Prognosis in virus-positive versus virus-negative.

A high level of heterogeneity was observed in the dataset (*I*^2^ = 47%, *P* = 0.05), so we used a random effects model to analyze the data. This showed that there was no significant difference in long-term prognosis of patients with virus-positive and virus-negative myocardial tissue (HR = 1.40, 95% CI = 0.93–2.12, *P* = 0.11; Figure 2).

### 3.3. Sensitivity Analysis

To examine the source of the heterogeneity in the dataset, we performed a sensitivity analysis. We found that the removal of Figulla et al.'s study [8] caused the heterogeneity to decrease from *I*^2^ = 47% (*P* = 0.05) to *I*^2^ = 22% (*P* = 0.25) and led to a significant difference in the long-term prognosis of the virus-positive and virus-negative groups (HR = 1.52, 95% CI = 1.08–2.13, *P* = 0.02, *n* = 9). Therefore, we performed a subgroup analysis to further explore the source of heterogeneity.

### 3.4. Subgroup Analysis

#### 3.4.1. Specific vs. Nonspecific Treatment

**Figure 3:**
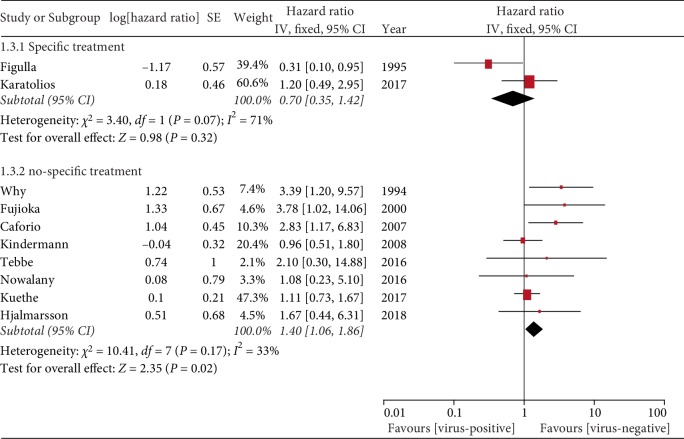
Specific treatment.

In Figulla et al.'s study [9], four patients in the virus-positive group had progressive deterioration of cardiac function but this improved after the administration of interferon alpha, and the same treatment was also used by Karatolios et al. [15]. Therefore, subgroup analysis was performed for this treatment. We found that in the absence of specific treatment, the virus-negative group was associated with a better prognosis than the virus-positive group (HR = 1.40, 95% CI = 1.06–1.86, *P* = 0.002; Figure 3).

#### 3.4.2. Polymerase Chain Reaction (PCR) vs. NonPCR Technology

**Figure 4:**
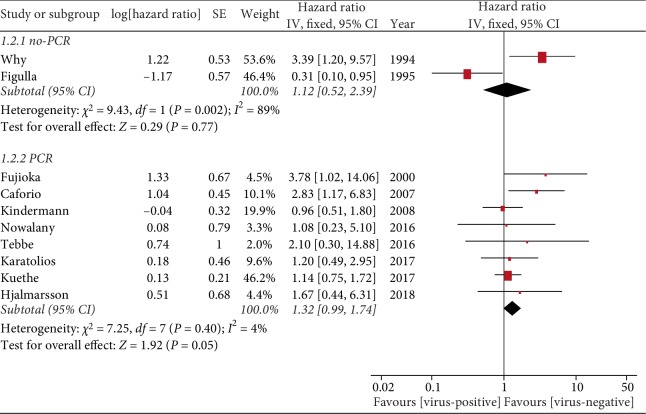
Virus detection methods.

Neither Why et al. [8] nor Figulla et al. [9] used PCR technology in the detection of viruses in the myocardial tissue, instead using molecular hybridization and in situ hybridization, respectively. By contrast, the other eight studies used more sensitive PCR techniques to detect viral DNA or RNA. However, we found that while the use of PCR technology could explain some of the heterogeneity, it did not affect the overall outcome (HR = 1.32, 95% CI = 0.99–1.74, *P* = 0.05; Figure 4).

#### 3.4.3. Left Ventricular vs. Right Ventricular EMB

**Figure 5:**
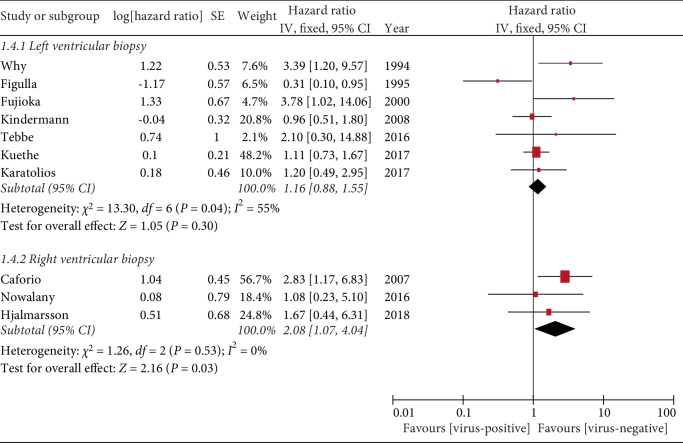
Left and right ventricular myocardial biopsy.

Seven of the studies carried out left ventricular EMB, while three performed right ventricular EMB. Among the former studies, only Kindermann et al. [12] performed the puncture under cardiovascular magnetic resonance imaging (CMR) and echocardiography. The subgroup analysis indicated that virus-negative right ventricular tissue was a protective factor for a good prognosis (HR = 2.08, 95% CI = 1.07–4.04, *P* = 0.03; Figure 5).

#### 3.4.4. Myocarditis and Dilated Cardiomyopathy

**Figure 6:**
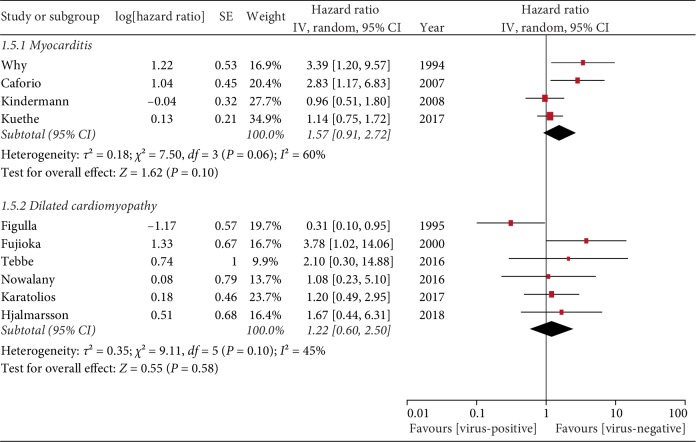
Myocarditis and dilated cardiomyopathy.

Myocarditis is generally considered to be one of the causes of progression to dilated cardiomyopathy. The preliminary diagnosis of the population in this study includes myocarditis and dilated cardiomyopathy. We try to group these two diagnoses and have a subsequent meta-analysis to find out the relationship between different prognosis and myocardial viral infection. The results showed that the prognosis was not related to whether the myocardium was infected with virus in neither myocarditis group nor dilated cardiomyopathy group. (Myocarditis group HR = 1.57, 95% CI = 0.91–2.72, *P* = 0.10; Dilated Cardiomyopathy HR = 1.22, 95% CI = 0.60–2.50, *P* = 0.58; Figure 6).

### 3.5. Publication Bias

**Figure 7:**
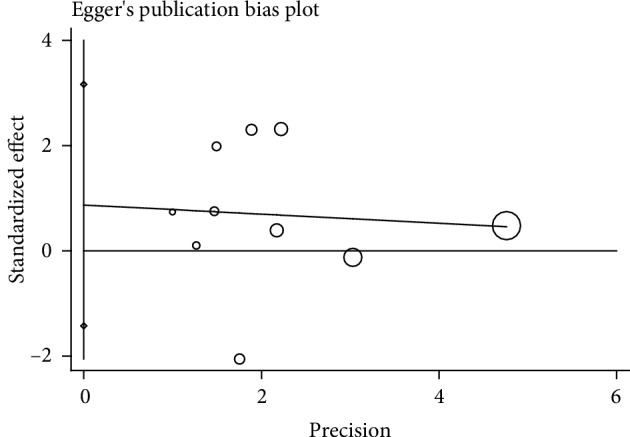
Publication bias.

We found that there was no publication bias using Egger's test (*P* = 0.407; Figure 7 and Table 4).

## 4. Discussion

In this meta-analysis, data from 10 cohort studies that included a total of 1006 patients with suspected myocarditis or dilated cardiomyopathy who underwent EMB were used to compare the long-term prognosis of patients with virus-positive and virus-negative myocardial tissue. The pooled results suggested that virus-positive patients did not have a worse prognosis than virus-negative patients, which is similar to the findings of most cohort studies. However, we believed that these results were not entirely reliable due to the high level of heterogeneity in the dataset (although the random effects model was used). Confirming this, a subgroup analysis suggested that virus-positive patients with suspected myocarditis and cardiomyopathy may have a worse prognosis where no specific treatment is used or right ventricular EMB is performed.

Figulla et al. [9] and Karatolios et al. [15] used specific treatments on their patients during their studies [antiviral therapy as an antiviral treatment (e.g., intravenous immunoglobulin, interferon) for virus-positive patients and an immunosuppressive drug for virus-negative patients]. In a 2016 meta-analysis of nine randomized controlled trial studies it was found that patients who were administered a specific treatment showed a significant improvement in cardiac function compared with those who were given a placebo (difference = 0.10, 95% CI = 0.00–0.21), but there was no significant difference in mortality or heart transplantation between the two groups (odds ratio = 1.33, 95% CI = 0.77–2.31) [18]. Therefore, specific treatment may have been one source of the heterogeneity in the present study.

Subgroup analysis also indicated that the detection of virus-positive tissues in a right ventricular biopsy was associated with a worse prognosis. Yilmaz et al. [19, 20] previously found that biventricular biopsy has a higher diagnostic value for myocarditis and cardiomyopathy than a selective single-ventricular biopsy (*P* < 0.001) and that in the case of biventricular biopsy, the left ventricular tissue appeared to be more diagnostically relevant than the right ventricular tissue (18.7% vs. 7.9%, respectively; *P* = 0.002). None of the studies that were included in this meta-analysis undertook biventricular biopsies. Furthermore, three studies undertook right ventricular biopsies and two of the seven studies that undertook left ventricular biopsies included specific treatment, which may have had confounding effects. Therefore, further research is required to determine whether right ventricular biopsy is more valuable for judging the prognosis.

Eight of the studies used PCR to detect viruses. However, Why et al. [8] and Figulla et al. [9] used the less sensitive methods of molecular hybridization and in situ hybridization. The inclusion of these studies in the meta-analysis caused the heterogeneity to reach 47%. However, the detection method was not found to have a significant effect on the results in the subgroup analysis.

This meta-analysis had the following limitations: (1) subjects who had received a heart transplantation or who were younger than 18 years of age were excluded from the analysis; (2) all of the studies included in the analysis were cohort studies, some sample sizes were small, and a loss of bias was unavoidable; (3) no-transplant survival was used as the observed outcome, with studies that included cardiac function and ventricular size changes as endpoints not being included; (4) nonenglish literature was excluded from the analysis and most of the research data came from European populations, with only one Asian population from Japan being included; (5) none of the studies conducted a second EMB before the end of the follow-up period so it was not possible to know whether the virus persisted in the myocardium and its effect on function; and (6) the follow-up times varied substantially between the studies. In addition, it should be noted that Why et al.'s study [8] included a 6-year-old child. However, we believe that this will not have had a significant impact on the results of the study and that there may have been a greater bias had it been excluded. Furthermore, studies involving cardiac amyloidosis were not included in the present analysis because of their poor prognosis and the fact that they are prone to false positives [20, 21].

Few studies have investigated the impact of myocardial viruses on survival prognosis to date. Therefore, larger, multicenter cohort studies are required to gain a better understanding of this.

## 5. Conclusions

In conclusion, the presence of a virus in the myocardium appears to have no effect on the long-term prognosis of patients with suspected myocarditis or dilated cardiomyopathy. However, subgroup analysis showed that this was a risk factor for poor prognosis in patients who did not receive specific treatment or who underwent right ventricular biopsy, suggesting that active antiviral therapy may improve the prognosis of virus-positive patients with cardiomyopathy. At the same time, an early myocardial biopsy to rule out the presence of viral infections in the myocardium will help to assess the prognosis and adjust the treatment strategies when considering myocarditis or cardiomyopathy in the case of unexplained heart failure.

## Figures and Tables

**Table 1:** Detailed characteristics of studies included in the meta-analysis.

Study	Country	Study period	Follow period (months)	Patients	Mean age	Diagnosis	Virus	Method	Study outcome
Why, H. J. F. et al. 1994	United Kingdom	1985–1989	11–50	Virus+: 40	44.9	MC/DCM	EV	Molecular hybridization	Virus-positive has bad prognosis
				Virus−: 76					
Figulla, H. R. et al. 1995	Germany	1987–1992	25.8 ± 13.7	Virus+: 20	48.2	IDCM	EV	In situ hybridization	Virus-positive has better prognosis
				Virus−: 57					
Fujioka, et al. 2000	Japan	1997–1998	6–12	Virus+: 9	49 ± 18	IDCM	EV	PCR	Virus-positive has bad prognosis
				Virus−: 17					
Caforio, A. L. P. et al. 2007	Italy	1992–2005	10–54	Virus+: 31	36 ± 18	AMC/BMC	HCV/EV/PVB19/ADV/EBV/HSV/CMV/MUMPS	PCR	Virus-positive has bad prognosis
				Virus−: 89					
Kindermann, I. et al. 2008	Germany	1994–2007	59 ± 42	Virus+ : 79	42 ± 15	Suspected viral myocarditis	EV/PVB19/ADV/EBV/HHV6	PCR	Survival NO difference
				Virus−: 101					
Nowalany-Kozielska, E. et al. 2016	Poland	2004–2007	10.8–61.2	Virus+: 32	44.9 ± 10.7	DCM	HCV/CVB/PVB19/CMV	PCR	Survival NO difference
				Virus−: 10					
Tebbe, U. et al. 2016	Germany	2003–2013	120	Virus+: 17	54	CM	HCV/EV/HHV6/PVB19/ADV/EBV/INFAB/HSV/VZV	PCR	Survival NO difference
				Virus−: 40					
Karatolios, K. et al. 2017	Germany	2004–2008	58.2 ± 19.8	Virus+: 16	51.1 ± 11.6	DCM	PVB19/CMV/HSV	PCR	Survival NO difference
				Virus−: 39					
Kuethe, F. et al. 2017	Germany	1997–2008	120	Virus+: 167	47.7 ± 12.6	CHF/MC/DCM	PVB19/EV/ADV/	PCR/RT-PCR	Survival NO difference
				Virus−: 126					
Hjalmarsson, C. et al. 2019	Sweden		112 ± 57	Virus+: 29	47 ± 12	IDCM	PVB19	PCR	Survival NO difference
				Virus−: 11					

MC: myocarditis, CHF: congestive heart failure, CM: cardiomyopathy, DCM/iDCM: dilated/idiopathic dilated cardiomyopathy, VZV: varicella-zoster virus, MUMPS: mumps virus, HSV: herpes simplex virus, EBV: epstein–Barr virus, HHV6: human herpes virus 6, ADV: adenovirus, INFA/B: InfluenzaA/B, CMV: cytomegalovirus, HCV: hepatitis C virus, PVB19: parvovirus-B19, PCR: polymerase chain reaction, RT-PCR: reverse transcription-polymerase chain reaction.

**Table 2:** Baseline data for studies included in meta-analysis.

Study	Arrhythmia∆	Echocardiography			Cardiac index (L/min/m2)	Heart failure duration (months)	New York Heart Association (NYHA)			
		LVEF (%)	LVEDD (mm)	LVEDP (mm Hg)			I	II	III	IV
Why, H. J. F. et al. 1994	Virus+: 8	Virus+: 38.9 ± 18.0	-	Virus+: 18.2 ± 9.8	-	Virus+: 7.8 ± 9.6	Virus+: 0	Virus+: 9	Virus+: 16	Virus+: 16
	Virus−: 22	Virus−: 36.2 ± 17.0		Virus−: 19.9 ± 10.0		Virus−: 14.9 ± 19.0	Virus−: 1	Virus−: 21	Virus−: 33	Virus−: 24
Figulla, H. R. et al. 1995	Virus+: 7	Virus+: 35	Virus+: 66	-	-	25	Virus+: 20	Virus+: 45	Virus+: 30	Virus+: 5
	Virus−: 8	Virus−: 34	Virus−: 64				Virus−: 16	Virus−: 47	Virus−: 35	Virus−: 2
Fujioka, S. et al. 2000	-	Virus+: 17.8 ± 6.6	Virus+: 80.4±7.9	-	-	-	0	0	Virus+: 2	Virus+: 7
		Virus−: 18.4 ± 7.4	Virus−: 79.9 ± 11.1						Virus−: 5	Virus−: 12
Caforio, A. L. P. et al. 2007	22	Virus+:38 ± 14	-	12	Virus+: 2.9		80	27	56	11
		Virus−: 45 ± 14			Virus−: 3.2					
Kindermann, I. et al. 2008	-	37.7 ± 18.5	36.2 ± 6.90	15.6 ± 7.40	-	-	39	52	73	17
Nowalany-Kozielska, E. et al. 2016	5	Virus+: 36.3 ± 14.7	Virus+: 59.6 ± 12.9	-		19.2 ± 6.4	Average NYHA:			
		Virus−: 37.2 ± 12.2	Virus−: 57.9 ± 11.1				Virus+: 1.9 ± 0.8			
							Virus−: 2.0 ± 0.8			
Tebbe, U. et al. 2016	-	50	-	-	-	-	-	-	-	-
Karatolios, K. et al. 2017	-	29.2 ± 8.5	70.1 ± 9.2	19.2 ± 8.8	-	-	4	23	25	3
Kuethe, F. et al. 2017	-	33.3 ± 13.5	63.6 ± 9.0	20.9 ± 9.2	2.1 ± 0.8	-	48	15	125	30
Hjalmarsson, C. et al. 2019	-	Virus+: 27 ± 13	-	-	Virus+: 2.3 ± 0.86	Virus+: 31 ± 15	Virus+: 3	Virus+: 9	Virus+: 10	Virus+: 7
		Virus−: 26 ± 12			Virus−: 2.0 ± 0.49	Virus−: 7 ± 8	Virus−: 0	Virus−: 4	Virus−: 5	Virus−: 2

∆: Atrial fibrillation and arrhythmia of nonsinus rhythm, LVEF: left ventricular ejection fraction, LVEDD: left ventricular end-diastolic dimension, LVEDP: left ventricular end-diastolic pressure.

**Table 3:** Assessment of the cohort studies by Newcastle-Ottawa scale.

Study (year)	Selection				Comparability	Outcome			Total scores
	Exposed cohort	Nonexposed cohort	Ascertainment of exposure	Outcome of interest		Assessment of outcome	Length of follow-up	Adequacy of follow-up	
Why, H. J. F. et al. 1994	★	★	★	★	★★	★	★	★	9
Figulla, H. R. et al. 1995	★	★	★	★	★☆	★	★	☆	7
Fujioka, et al. 2000	★	★	★	★	★★	★	☆	★	8
Caforio, A. L. P. et al. 2007	★	★	★	☆	★☆	★	★	★	7
Kindermann, I. et al. 2008	★	★	★	★	★☆	★	★	★	8
Nowalany-Kozielska, E. et al. 2016	★	★	★	☆	★★	★	★	★	8
Tebbe, U. et al. 2016	★	★	★	★	★★	★	★	☆	8
Karatolios, K. et al. 2017	★	★	★	★	★☆	★	★	☆	7
Kuethe, F. et al. 2017	★	★	★	★	★★	★	★	☆	8
Hjalmarsson, C. et al. 2019	★	★	★	★	★★	★	★	★	9

**Table 4:** The value in publication bias.

Std_Eff	Coef.	Std. Err.	*t*	*P* > |*t*|	[95% Conf. Interval]	
Slope	−0.08622	0.423287	−0.20	0.844	−1.062322	0.889882
Bias	0.86987	0.994077	0.88	0.407	−1.422481	3.162211

## Data Availability

The data used to support the findings of this study are available from the corresponding author upon request.
